# Influence of delivery characteristics and socioeconomic status on giving birth by caesarean section – a cross sectional study during 2000–2010 in Finland

**DOI:** 10.1186/1471-2393-14-120

**Published:** 2014-03-31

**Authors:** Sari Räisänen, Mika Gissler, Michael R Kramer, Seppo Heinonen

**Affiliations:** 1 Department of Epidemiology, Rollins School of Public Health, Emory University, 1518 Clifton Road NE, Atlanta, GA, USA; 2 Department of Obstetrics and Gynaecology, Kuopio University Hospital, P.O. Box 100, Kys, Kuopio, FI 70029, Finland; 3 National Institute for Health and Welfare (THL), P.O. Box 30, Paciuksenkatu 21, Helsinki, FI 00271, Finland; 4 Nordic School of Public Health, Gothenburg, Sweden; 5 School of Medicine, University of Eastern Finland, P.O. Box 1627, Kuopio, FI 70211, Finland

**Keywords:** Birth, Caesarean section, Childbirth, Population register, Register, Socioeconomic status

## Abstract

**Background:**

Caesarean section (CS) rates especially without medical indication are rising worldwide. Most of indications for CS are relative and CS rates for various indication**s** vary widely. There is an increasing tendency to perform CSs without medical indication on maternal request. Women with higher socioeconomic status (SES) are more likely to give birth by CS. We aimed to study whether giving birth by CS was associated with SES and other characteristics among singleton births during 2000–2010 in Finland with publicly funded health care.

**Methods:**

Data were gathered from the Finnish Medical Birth Register. The likelihood of giving birth by CS according to CS type (planned and non-planned), parity (nulliparous vs. multiparous), socio-demographic factors, delivery characteristics and time periods (2000–2003, 2004–2007 and 2008–2010) was determined by using logistic regression analysis. SES was classified as upper white collar workers (highest SES), lower white collar workers, blue collar workers (lowest SES), others (all unclassifiable cases) and cases with missing information.

**Results:**

In total, 19.8% (51,511 of 259,736) of the nulliparous women and 13.1% (47,271 of 360,727) of the multiparous women gave birth by CS. CS was associated with several delivery characteristics, such as placental abruption, placenta previa, birth weight and fear of childbirth, among both parity groups. After adjustment, the likelihood of giving birth by planned CS was reduced by 40% in nulliparous and 55% in multiparous women from 2000–2003 to 2008–2010, whereas the likelihood of non-planned CSs did not change. Giving birth by planned and non-planned CS was up to 9% higher in nulliparous women and up to 17% higher in multiparous women in the lowest SES groups compared to the highest SES group.

**Conclusions:**

Giving birth by CS varied by clinical indications. Women with the lowest SES were more likely to give birth by CS, indicating that the known social disparity in pregnancy complications increases the need for operative deliveries in these women. Overall, the CS policy in Finland shows favoring a trial of labor over planned CS and reflects no inequity in healthcare services.

## Background

Caesarean section (CS) rates vary substantially between countries, especially between low-income and high-income countries, indicating a great disparity in the availability of this life-saving obstetric procedure across the world [1-3]. The World Health Organization (WHO) has recommended an optimal CS rate of 15% based on the CS rates of countries with the lowest maternal and neonatal mortality rates [4]. CS rates have been constantly rising in many developed countries, such as the European countries [5], especially due to the increasing tendency to perform CSs without medical indication on maternal request [6,7]. In 2010, among the European countries, CS rates varied from 52.2% in Cyprus to 14.8% in Iceland. Only The Netherlands, Slovenia, Finland, Sweden, Iceland, and Norway had CS rates below 20% [5]. However, CS rates in Europe did not correlate with national wealth, as measured by the gross domestic product (http://epp.eurostat.ec.europa.eu/statistics_explained/index.php/GDP_per_capita,_consumption_per_capita_and_price_level_indices). The major indications for CS are repeated caesarean section, dystocia, breech presentation, foetal conditions (such as macrosomia, prematurity, anomalies or abnormal lie), foetal distress, multiple pregnancies and maternal conditions (such as antepartum haemorrhage, diabetes and preeclampsia) [8]. However, most of the indications are relative and CS rates for various indications vary widely [8]. Previous population based studies from Australia, Canada and UK reported higher prevalence of CS performed due to repeated CS [9-11], dystocia [11] and elective prelabor CS, especially for nulliparous women [12]. A few previous studies have demonstrated differences in CS rates between socioeconomic status (SES) groups within countries. Women with low SES or living in deprived areas were less likely to give birth by CS compared to women with high SES or living in affluent areas [13,14], even in countries with publicly funded healthcare [15,16], indicating that inequity may exist in some healthcare services. On the other hand, a large population based study from the Norway reported that women with the lowest level of education were more likely to give birth by CS [17].

In Finland, previous studies have reported SES disparities in amenable deaths [18] and invasive cardiac procedures [19,20], showing that women with the highest SES have the lowest risk. Further, women with the highest SES were more likely to undergo in vitro fertilization in Finland [21-23]. In Finland, practically all pregnant women give birth in public hospitals with free-access, and private delivery care services are not provided. The aim of the present study was to explore whether giving birth by planned and non-planned CS was associated with SES and other characteristics among singleton pregnancies during 2000–2010 in Finland.

## Methods

### Data and population

The study population included all women with singleton births (*N* = 620,463) during 2000–2010 in Finland; multiple births (*n* = 19,305) were excluded. Data were gathered retrospectively from the Finnish Medical Birth Register (MBR) and included information on socio-demographic, pregnancy and delivery characteristics, and diagnoses on all live births or stillbirths (after the 22nd gestational week or weighing 500 g or more) during the first seven days after birth. Information on maternal diseases and reproductive risk factors, such as preeclampsia, diabetes mellitus, gestational diabetes, placental abruption, depression and fear of childbirth, was gathered based on the International Classification of Diseases (ICD-10) codes contained in the Hospital Discharge Register (HDR) and linked with the MBR data by using parturients’ encrypted personal identification numbers. Both national health registers (established in 1987 and 1967, respectively) are currently maintained by the National Institute for Health and Welfare (THL).

### Variables and definitions

Women were classified into two groups based on the number of prior childbirths: nulliparous (no prior childbirths) and multiparous women (one or more prior childbirths). Mode of delivery was defined either as vaginal birth, including spontaneous vaginal, breech, instrumental (forceps or vacuum assisted) or CS (planned or non-planned). Maternal age was classified as less than 20, 20–29, 30–39 and 40 years or more (advanced age). Birth weight was classified as less than 3000, 3000–3499, 3500–3999 and 4000 grams or more. Women were also grouped based on self-reported smoking habits during pregnancy: non-smoking, quitted smoking during the first trimester or continued smoking after the first trimester, i.e., smoking and missing information. Marital status was classified as either married (including unmarried women living with a partner) or single.

SES was categorized based on Finland’s National Classification of Occupations [24], which follows international recommendations. SES was categorized based on maternal occupation at birth, yielding five groups: upper white-collar workers, such as physicians and lawyers; lower white-collar workers, such as nurses and secretaries; blue-collar workers, such as cooks and cleaners; others; and missing information, as categorized and published elsewhere [25]. ‘Others’ included all cases with unclassifiable occupations, such as entrepreneurs, students, retired, unemployed and housewives. The category with missing information on SES comprised 17% (*n* = 105,472) of all cases.

Information on preeclampsia, diabetes mellitus, gestational diabetes, placental abruption and fear of childbirth was defined according to ICD-10 codes gathered from the HDR. Information on prior miscarriages and terminations was dichotomous (yes or no). In vitro fertilization (IVF) included intracytoplasmic sperm injection (ICSI) and frozen embryo transfers (FET). Body mass index (BMI) gathered since 2004 was calculated by dividing body weight in kilograms by the squared height in meters (kg/m^2^). The study period was divided into three time periods (2000–2003, 2004–2007 and 2008–2010) to evaluate secular trends.

### Ethical approval

Permission to use the confidential register data in this study was granted on 16th February, 2012 by the THL in Finland. THL also approved the study (Reference number 1749/5.05.00/2011).

### Statistical analyses

Bivariable analyses were performed separately for nulliparous and multiparous women with different prevalence of CS (*p* ≤ 0.001). Differences between the groups (nulliparous and multiparous women giving birth with and without CS, any type) were evaluated by chi square test for dichotomous and categorical variables, and Student’s *t*-test for continuous variables.

To test whether there were differences in the prevalence of CS births (separately for planned and non-planned) between SES groups and other characteristics, we performed unconditional logistic regression analysis (backward elimination), using women with vaginal birth as a reference group in both parity groups. Possible confounders (maternal age, birth weight, foetal sex, smoking status, marital status, SES, induction, preeclampsia, gestational diabetes, maternal diabetes mellitus, fear of childbirth, placental abruption, placenta previa, IVF, prior terminations, prior miscarriages, prior CS and time period) were selected based on bi-variable analyses (*p* < 0.1). In addition, we studied the contribution of demographics and delivery characteristics on the odds ratio (OR) of SES for CS births (separately for planned and non-planned) according to parity (nulliparous and multiparous women), using women with vaginal birth as a reference group. Each variable was added separately to Model 2 (adjusted by maternal age), prior CS birth (in multiparous women) (Model 3), birth weight (Model 4), smoking (Model 5), and the full model (Model 6, adjusted by age, prior CS births, birth weight and smoking). OR with 95% confidence intervals (CI) were calculated. The contribution of smoking to CS associated with SES was measured based on the percentage reduction in the OR of SES by using the formula (OR Model 2 – OR Model 5)/(OR Model 2 – 1). To avoid bias arising from missing information on SES, we performed further analyses by using multiple imputations. Differences were deemed to be significant if the *p*-value was less than 0.05. The data were analyzed using SPSS for Windows 19.0, Chicago, IL.

## Results

In total, 19.8% (51,511 of 259,736) of the nulliparous women and 13.1% (47,271 of 360,727) of the multiparous women with singleton births gave birth by CS during 2000–2010 in Finland (Table 1). The total CS rate was relatively constant during the 11-year study period among both parity groups. Women who gave birth by CS were in general older, gave birth more frequently to a male infant with lower mean gestational age and were more likely to have high SES (upper or lower white collar worker) than women who gave birth vaginally, regardless of parity. As expected, giving birth by CS was associated with several reproductive risk factors during index pregnancy, such as induction, preeclampsia, gestational diabetes, diabetes mellitus, IVF, placental abruption, placenta previa and fear of childbirth, as well as prior history of pregnancy terminations and miscarriages among both parity groups. Nulliparous women with infants weighing less than 3000 grams or at least 4000 grams and multiparous women with infants weighing less than 3000 grams were more likely to undergo CS. Multiparous women were more likely to give birth by CS if they had a prior history of CS birth.

**Table 1:** Demographics and delivery characteristics among singleton births according to caesarean section (CS) and parity during 2000–2010 in Finland

**Characteristic**	**Nulliparous, *n*** **= 259,736**		***p *** value*	**Multiparous, *n*** **= 360,727**		***p *** value*
	**CS, *n*** **= 51,511 (19.8%)**	**Vaginal birth, *n*** **= 208,225**		**CS, *n*** **= 47,271 (13.1%)**	**Vaginal birth, *n*** **= 313,456**	
Planned CS, *n* (% of CSs)	15,420 (29.9)	NA		26,987 (57.1)	NA	
Non-planned CS, *n* (% of CSs)	36,091 (70.1)	NA		20,284 (42.9)	NA	
Mean number of prior births (SD)	NA	NA		1.7 (1.3)	1.8 (1.5)	≤ 0.001
Mean maternal age, years (SD)	29.0 (5.5)	27.0 (5.1)	≤ 0.001	32.4 (5.1)	30.8 (5.0)	≤ 0.001
Maternal age, years %			≤ 0.001			≤ 0.001
< 20	3.5	6.6		0.2	0.4	
20-29	51.6	63.4		28.8	40.5	
30-39	41.4	28.8		62.8	54.6	
40 or more	3.5	1.2		8.2	4.5	
Mean gestational age, weeks (SD)	39.4 (2.4)	39.9 (1.7)	≤ 0.001	38.8 (2.3)	39.9 (1.6)	≤ 0.001
Mean birth weight, g (SD)	3398 (710)	3439 (500)	≤ 0.001	3492 (720)	3524 (509)	≤ 0.001
Birth weight, g%			≤ 0.001			≤ 0.001
< 3000	22.3	16.0		18.1	9.1	
3000-3499	29.8	37.9		28.2	29.1	
3500-3999	30.0	34.4		31.6	39.7	
4000 or more	17.9	11.7		22.1	22.1	
Male fetal sex %	53.8	50.6	≤ 0.001	52.4	51.0	≤ 0.001
Smoking status %			≤ 0.001			≤ 0.001
Non-smoking	80.9	80.3		82.8	84.2	
Quit smoking during 1st trimester	5.0	5.2		2.5	2.3	
Smoking after 1st trimester	11.7	12.6		11.0	10.6	
Missing information	2.4	2.0		3.7	2.9	
Married or living with a partner %	91.3	90.5	≤ 0.001	95.0	95.2	0.07
Socioeconomic status %			≤ 0.001			≤ 0.001
Upper white-collar workers	8.9	7.8		8.8	8.5	
Lower white-collar workers	34.2	31.3		38.6	36.3	
Blue-collar workers	13.9	13.7		15.5	15.0	
Others^a^	24.2	27.7		21.7	25.0	
Missing information	18.8	19.5		15.4	15.2	
Mean body mass index (SD)	24.8 (5.0)	23.5 (4.3)	≤ 0.001	25.9 (5.5)	24.4 (4.7)	≤ 0.001
Induction %	19.1	16.2	≤ 0.001	9.1	17.1	≤ 0.001
Preeclampsia %	3.3	0.3	≤ 0.001	4.3	1.1	≤ 0.001
Gestational diabetes %	11.3	6.7	≤ 0.001	19.7	12.1	≤ 0.001
Maternal diabetes mellitus %	9.1	4.7	≤ 0.001	15.7	8.9	≤ 0.001
In vitro fertilization (IVF) %	3.4	1.9	≤ 0.001	1.5	0.7	≤ 0.001
Placental abruption %	83.5	16.5	≤ 0.001	78.0	22.0	≤ 0.001
Placenta previa %	78.2	21.8	≤ 0.001	81.4	18.6	≤ 0.001
Fear of childbirth %	6.5	2.0	≤ 0.001	15.3	4.1	≤ 0.001
Prior terminations %	12.3	11.1	≤ 0.001	14.9	13.3	≤ 0.001
Prior miscarriages %	15.2	12.2	≤ 0.001	29.3	26.0	≤ 0.001
Prior caesarean section %	NA	NA		64.3	11.1	≤ 0.001
Time period %			0.18			≤ 0.001
2000-2003	19.7	80.3		13.3	86.7	
2004-2007	19.8	80.2		13.2	86.8	
2008-2010	20.0	80.0		12.7	87.3	

^a^Others comprised entrepreneurs, students, retired women, unemployed women, housewives and all unclassifiable cases.

*Chi-square or Student’s *t*–test, SD = standard deviation, NA = not applicable.

After adjustment for case-mix in logistic regression analysis, the prevalence of planned CS births in nulliparous women was 9% (adjusted odds ratio (aOR) 1.09, 95% confidence interval (CI) 1.03-1.11) higher in lower white collar workers, and the prevalence of non-planned CS birth was 7% (aOR 1.07, 95% CI 1.01-1.13) higher in blue collar workers compared to upper white collar workers (Table 2). In multiparous women, the prevalence of planned CS was 17% (aOR 1.17, 95% CI 1.11-1.24) higher in lower white collar workers and 16% (aOR 1.16, 95% CI 1.09-1.24) higher in blue collar workers compared to upper white collar workers (Table 2), whereas the prevalence of non-planned CS was 15% (aOR 1.15, 95% CI 1.07-1.23) higher in blue collar workers compared to upper white collar workers. Differences between other SES groups were non-significant regardless of parity and CS type.

**Table 2:** Adjusted odds ratios (aORs) of planned and non-planned caesarean section (CS) births according to parity during 2000–2010 in Finland, using women with vaginal birth as a reference group in all analyses (logistic regression analysis)

**Characteristic**	**Nulliparous women**		**Multiparous women**	
	**Planned CS, *n*** **= 14,514**	**Non-planned CS, *n*** **= 33,873**	**Planned CS, *n*** **= 26,132**	**Non-planned CS, *n*** **= 19,555**
	**aOR (95% CI)**	**aOR (95% CI)**	**aOR (95% CI)**	**aOR (95% CI)**
Maternal age (years)				
< 20	1	1	1	1
20-29	1.35 (1.23-1.49)	1.56 (1.46-1.67)	1.34 (0.97-1.85)	1.08 (0.81-1.44)
30-39	2.39 (2.17-2.64)	2.54 (2.37-2.72)	1.94 (1.40-2.69)	1.37 (1.03-1.83)
40 or more	4.85 (4.23-5.55)	4.14 (3.75-4.58)	2.74 (1.97-3.81)	1.83 (1.36-2.46)
Birth weight (g)				
<3000	1.56 (1.48-1.64)	1.69 (1.64-1.75)	1.49 (1.42-1.57)	3.49 (3.33-3.65)
3000-3499	1.20 (1.15-1.26)	1	1.30 (1.26-1.35)	1.09 (1.05-1.14)
3500-3999	1	1.27 (1.23-1.31)	1	1
4000 or more	1.47 (1.39-1.56)	2.24 (2.16-2.33)	1.13 (1.06-1.21)	1.46 (1.40-1.52)
Male fetal sex	0.94 (0.91-0.98)	1.20 (1.67-1.22)	1.04 (1.01-1.07)	1.20 (1.17-1.24)
Smoking status				
Non-smoking	1	1	1	1
Quit smoking during 1st trimester	1.06 (0.98-1.13)	1.07 (1.01-1.13)	1.07 (0.97-1.18)	1.18 (1.07-1.31)
Smoking after 1st trimester	0.94 (0.89-1.00)	1.10 (1.06-1.15)	1.01 (0.96-1.06)	1.00 (0.95-1.06)
Missing information	1.36 (1.21-1.52)	1.15 (1.06-1.25)	1.41 (1.30-1.53)	1.17 (1.07-1.28)
Single (ref married/living with a partner)	0.93 (0.87-0.99)	0.99 (0.95-1.03)	1.02 (0.95-1.10)	1.12 (1.04-1.20)
Socioeconomic status				
Upper white collar workers	1	1	1	1
Lower white collar workers	1.09 (1.02-1.16)	1.05 (1.00-1.10)	1.17 (1.11-1.24)	1.07 (1.00-1.11)
Blue collar workers	1.03 (0.95-1.11)	1.07 (1.01-1.13)	1.16 (1.09-1.24)	1.15 (1.07-1.23)
Others^a^	0.95 (0.89-1.02)	0.93 (0.88-0.97)	0.98 (0.92-1.04)	0.92 (0.87-0.98)
Missing information	0.96 (0.89-1.03)	1.01 (0.96-1.06)	1.13 (1.06-1.21)	1.08 (1.01-1.15)
Induction	NA	1.64 (1.59-1.68)	NA	0.97 (0.94-1.01)
Preeclampsia	6.41 (5.53-7.44)	11.77 (10.62-13.04)	1.04 (0.96-1.14)	2.12 (1.95-2.30)
Gestational diabetes	0.55 (0.49-0.62)	1.34 (1.26-1.43)	1.07 (1.01-1.15)	1.39 (1.30-1.48)
Maternal diabetes mellitus	3.95 (3.51-4.46)	1.14 (1.06-1.23)	1.66 (1.54-1.78)	1.06 (0.98-1.15)
Fear of childbirth	7.99 (7.53-8.48)	1.58 (1.48-1.70)	5.05 (4.83-5.28)	1.67 (1.58-1.78)
Placental abruption	NA	29.42 (24.22-35.74)	NA	61.78 (52.87-72.20)
Placenta previa	25.35 (20.56-31.25)	8.05 (6.46-10.04)	39.65 (32.73-48.04)	22.41 (18.46-27.20)
In vitro fertilization (IVF)	1.25 (1.13-1.38)	1.25 (1.16-1.34)	1.49 (1.30-1.72)	1.86 (1.63-2.12)
Prior terminations	1.02 (0.97-1.08)	1.09 (1.05-1.13)	1.10 (1.05-1.15)	1.10 (1.05-1.15)
Prior miscarriages	1.16 (1.10-1.22)	1.12 (1.08-1.16)	0.99 (0.96-1.03)	1.00 (0.96-1.03)
Prior caesarean section	NA	NA	20.60 (19.97-21.24)	8.32 (8.06-8.60)
Time period				
2000-2003	1.40 (1.34-1.47)	0.94 (0.92-0.97)	1.55 (1.49-1.61)	1.02(0.98-1.06)
2004-2007	1.21 (1.16-1.27)	0.95 (0.92-0.98)	1.22 (1.18-1.27)	1.03 (0.99-1.07)
2008-2010	1	1	1	1

^a^Others comprised entrepreneurs, students, retired women, unemployed women, housewives and all unclassifiable cases, NA = not applicable.

Placenta previa was the strongest risk factor for planned CS and placental abruption was the strongest risk factor for non-planned CS, regardless of parity. Other delivery characteristics associated with planned CS birth were advanced maternal age (≥ 40 years), a birth weight of less than 3000 grams, a birth weight of 4000 grams or more, maternal diabetes mellitus, fear of childbirth and IVF, regardless of parity. Delivery characteristics associated with non-planned CS birth were advanced maternal age, a birth weight of less than 3000 grams, a birth weight of 4000 grams or more, preeclampsia, gestational diabetes, fear of childbirth, IVF and prior terminations, regardless of parity. Further, in nulliparous women, an increased prevalence of non-planned CS birth was associated with smoking and induction of labor. In multiparous women, prior CS birth was associated with an increased risk of both CS types.

After adjustment for case-mix, the prevalence of planned CS appeared to decrease by 40% in nulliparous women and by 55% in multiparous women from 2000–2003 to 2008–2010, whereas differences in non-planned CSs between the time periods were non-significant. The same logistic regression analyses were performed for both groups of women using multiple imputations for the missing data, but the results did not change (data not shown).

Table 3 presents crude and adjusted ORs of SES for CS birth (separately for planned and non-planned CSs) according to parity and different confounders. Delivery characteristics were added separately to Model 2 (adjusted for maternal age) to evaluate the contribution of each characteristic to the ORs of SES for CS birth. It appeared that maternal age made a major contribution and maternal smoking during pregnancy a moderate contribution to the prevalence of both CS types associated with SES. Further, the contribution made by the birth weight was minor. In nulliparous women, 21.4% of the differences in planned CS birth prevalence and 17.4% of the differences in non-planned CS birth prevalence between blue collar workers and upper white collar workers could be explained by smoking during pregnancy (percentage reduction in OR of SES-CS between Model 2 and Model 5 in Table 3). In multiparous women, 18.8% and 19.2% of the difference between blue collar workers and upper white collar workers in planned and non-planned CS births, respectively, could be explained by smoking during pregnancy.

**Table 3:** Odds ratios (ORs) of planned and non-planned caesarean section (CS) births associated with socioeconomic status (SES) after adjustment for delivery characteristics according to parity during 2000–2010 in Finland

	**Model 1 crude**	**Model 2**	**Model 3**	**Model 4**	**Model 5**	**Model 6**
		**Adjusted by maternal age**	**Adjusted by Model 2 + prior CS**^**a**^	**Adjusted by Model 2 + birth weight**	**Adjusted by Model 2 + smoking**	**Adjusted by Model 2+ prior CS**^**a**^**, birth weight and smoking**
**Planned CS**	**OR (95% CI)**	**OR (95% CI)**	**OR (95% CI)**	**OR (95% CI)**	**OR (95% CI)**	**OR (95% CI)**
**Nulliparous women**						
SES						
Upper white collar workers	1	1	NA	1	1	1
Lower white collar workers	0.93 (0.87-0.98)	1.13 (1.07-1.21)		1.13 (1.06-1.20)	1.13 (1.06-1.20)	1.12 (1.05-1.19)
Blue collar workers	0.79 (0.74-0.85)	1.14 (1.06-1.22)		1.13 (1.05-1.21)	1.11 (1.03-1.20)	1.11 (1.03-1.19)
Others^b^	0.74 (0.70-0.80)	1.04 (0.97-1.11)		1.04 (0.97-1.10)	1.03 (0.97-1.10)	1.03 (0.97-1.10)
Missing information	0.76 (0.71-0.81)	1.01 (0.95-1.08)		1.00 (0.94-1.07)	1.00 (0.93-1.07)	0.99 (0.93-1.06)
**Multiparous women**						
SES						
Upper white collar workers	1	1	1	1	1	1
Lower white collar workers	1.03 (0.98-1.08)	1.15 (1.10-1.21)	1.17 (1.11-1.24)	1.15 (1.10-1.20)	1.14 (1.09-1.20)	1.16 (1.10-1.22)
Blue collar workers	0.96 (0.91-1.01)	1.16 (1.10-1.22)	1.18 (1.11-1.25)	1.14 (1.08-1.21)	1.13 (1.07-1.19)	1.14 (1.08-1.22)
Others^b^	0.83 (0.79-0.88)	1.00 (0.95-1.05)	1.02 (0.96-1.07)	0.99 (0.94-1.04)	0.99 (0.94-1.04)	1.00 (0.95-1.06)
Missing information	0.95 (0.90-1.00)	1.12 (1.06-1.18)	1.16 (1.09-1.23)	1.11 (1.05-1.17)	1.10 (1.05-1.17)	1.14 (1.07-1.21)
**Non-planned CS**	**OR (95% CI)**	**OR (95% CI)**	**OR (95% CI)**	**OR (95% CI)**	**OR (95% CI)**	**OR (95% CI)**
**Nulliparous women**						
SES						
Upper white collar workers	1	1	NA	1	1	1
Lower white collar workers	0.96 (0.92-1.00)	1.13 (1.08-1.18)		1.13 (1.08-1.18)	1.12 (1.07-1.17)	1.12 (1.07-1.17)
Blue collar workers	0.92 (0.87-0.96)	1.23 (1.17-1.29)		1.22 (1.16-1.29)	1.19 (1.13-1.25)	1.19 (1.13-1.25)
Others^b^	0.77 (0.73-0.80)	1.01 (0.97-1.06)		1.01 (0.96-1.05)	1.00 (0.96-1.05)	1.00 (0.96-1.05)
Missing information	0.87 (0.83-0.91)	1.10 (1.05-1.15)		1.10 (1.05-1.15)	1.09 (1.03-1.14)	1.08 (1.03-1.13)
**Multiparous women**						
SES						
Upper white collar workers	1	1	1	1	1	1
Lower white collar workers	1.04 (0.99-1.10)	1.14 (1.08-1.20)	1.15 (1.09-1.22)	1.12 (1.06-1.18)	1.12 (1.06-1.19)	1.12 (1.06-1.19)
Blue collar workers	1.09 (1.02-1.15)	1.26 (1.18-1.33)	1.29 (1.21-1.37)	1.20 (1.13-1.27)	1.21 (1.14-1.29)	1.22 (1.14-1.30)
Others^b^	0.87 (0.82-0.92)	1.00 (0.94-1.06)	1.01 (0.96-1.08)	0.97 (0.91-1.03)	0.98 (0.93-1.04)	0.98 (0.92-1.04)
Missing information	1.04 (0.98-1.10)	1.18 (1.11-1.25)	1.20 (1.12-1.28)	1.12 (1.06-1.19)	1.16 (1.09-1.23)	1.14 (1.06-1.21)

NA = not applicable, ^a^ Prior CS birth adjusted only in multiparous women.

^b^Others comprised entrepreneurs, students, retired women, unemployed women, housewives and all unclassifiable cases.

## Discussion

### Statement of principal findings

During the study period of 2000–2010 in Finland, the total CS rate in nulliparous women was 19.8% and 13.1% in multiparous women. Giving birth by CS varied by clinical indications reflecting equity in delivery care services. Giving birth by CS (planned or non-planned) was 7-9% more common in nulliparous women with lower SES compared to upper white collar workers (highest SES). Giving birth by CS (planned or non-planned) was 15-17% more common in multiparous women with lower SES compared to upper white collar workers. Maternal age made a major contribution to the variation in CS births between the SES groups, whereas the contribution of smoking during pregnancy was moderate. After adjustment for background factors, giving birth by planned CS reduced by 40% in nulliparous women and by 55% in multiparous women from 2000–2003 to 2008–2010, whereas differences in giving birth by non-planned CS were non-significant. These figures indicate that as a policy, trial of labor has been favored over planned CS.

### Strengths and weaknesses

The present study has several strengths. First, the data, covering the total population of women with singleton births during recent years, were gathered from two national health registers containing high quality data. Second, we were able to use a variety of characteristics as confounders in the analyses, and performed analyses separately for nulliparous and multiparous women with significantly different prevalence of CS. The most important limitation was that information on SES was missing in 17% of the cases. Information on SES is optional and an increasing number of women do not wish to provide this type of sensitive information. Based on our previous analyses and characteristics, we suggest that women with missing SES represented all SES categories. To reduce the bias caused by missing information on SES, we performed multiple imputations, but this did not change the results. Further, SES was solely based on maternal occupation at birth and we did not have information on education and household income. However, in Finland, these are known to correlate with occupation, which is therefore an appropriate indicator for studies on socioeconomic health disparity [26,27]. Further, we did not have information on spouses’ SES because of data protection issues. Another limitation was that the MBR does not include the actual diagnosis of CS with ICD-10 codes CS. Further, a proportion of the planned CSs performed out of office hours might have been classified as non-planned CS births.

### Meaning and implications of the study

In the present study, we found that giving birth by CS was strongly associated with delivery characteristics such as birth weight and reproductive risk factors such as advanced maternal age, placental abruption and placenta previa in a country with exclusively publicly funded delivery care services. Further, we found up to 9 and 17% higher prevalence of CS births (any type) in women with lower SES (lower white collar workers and blue collar workers) compared to women with the highest SES (upper white collar workers) for nulliparous and multiparous women, respectively. This finding was in accordance with a previous study that reported a parallel but constantly increasing SES disparity during 1967–2004 in Norway, which has a similar kind of welfare system to Finland [17]. In the present study, the SES disparity was smaller among nulliparous women than multiparous women. It might be speculated that the increased prevalence of CS births in the nulliparous women with lower SES compared to the highest SES might result in an increased SES disparity in subsequent births due to prior CS birth, which is a strong risk factor for CS in subsequent births, as reported by previous studies [28,29]. That might in part explain the constantly rising prevalence of CS births among women with the lowest education reported by a Norwegian study [17].

The higher prevalence of CS births among the women with lowest compared to highest SES might partially be explained by an increased risk of adverse outcomes, such as preterm birth and small for gestational age (SGA), as shown previously in Finland using the same data [30,31], particularly as adverse health behavior such as smoking is known to be strongly associated with SES [32]. In the present study, we observed an up to 21.4% and 19.2% difference in the prevalence of CS births between women with lower SES compared to the highest SES for nulliparous and multiparous women, respectively, which could be explained by smoking during pregnancy. High risk pregnancies, such as those exhibiting growth restriction, are more likely to result in other complications, such as foetal distress, that require CS birth. However, due to a lack of information on actual CS indications, we could not study that aspect. Furthermore, it might be speculated that a part of SES disparity in giving birth by CS might be explained by differences in delivery training. The results of the present study contradicted those obtained in previous studies, which indicated inequity in both the access to healthcare and healthcare services offered, with a positive social gradient in IVF services [21] and cardiac procedures [19,20] in Finland.

## Conclusions

We conclude that the mode of delivery is an important healthcare policy issue since the constantly rising prevalence of CS births, especially in medium- and high-income countries, does not seem to translate into better maternal and neonatal outcomes [2,29]. In the present study, using the most recent population based data, we detected a decreasing prevalence of planned CS births among singleton pregnancies in Finland with good perinatal outcomes assessed based on several quality indicators [5]. However, we observed a higher prevalence of CS births among women with lower SES compared to the highest SES, which probably reflects the known social gradient in pregnancy complications, and consequently increased need for operative deliveries in women with social deprivation. Overall, the CS policy in Finland shows no inequity in healthcare, which is an important quality indictor in publicly funded services.

## Abbreviations

aOR: Adjusted odds ratio; BMI: Body mass index; CI: Confidence interval; CS: Caesarean section; FET: Frozen embryo transfers; ICSI: Intracytoplasmic sperm injection; ICD: International classification of diseases; IVF: In vitro fertilization; OR: Odds ratio; SES: Socioeconomic status; THL: National Institute for Health and Welfare; WHO: World Health Organization.

## Competing interests

The authors declare that they have no competing interests.

## Authors’ contributions

SR, MG, MRK and SH participated in designing the study. SR managed the dataset and performed statistical analyses. MG, MRK and SH gave advice regarding the statistical analyses. All authors contributed to the interpretation of the results, as well as to the writing and editing of the manuscript. All authors read and approved the final manuscript.

## Pre-publication history

The pre-publication history for this paper can be accessed here:

http://www.biomedcentral.com/1471-2393/14/120/prepub
